# Reevaluating the role of platinum-based chemotherapy in the evolving treatment landscape for patients with advanced urothelial carcinoma

**DOI:** 10.1093/oncolo/oyae215

**Published:** 2024-08-21

**Authors:** Tian Zhang, Alan Tan, Amishi Y Shah, Gopa Iyer, Valerie Morris, Sébastien Michaud, Srikala S Sridhar

**Affiliations:** Department of Internal Medicine, Simmons Comprehensive Cancer Center, UT Southwestern Medical Center, Dallas, TX, United States; Department of Medicine, Vanderbilt University Medical Center, Nashville, TN, United States; Department of Genitourinary Medical Oncology, Division of Cancer Medicine, The University of Texas MD Anderson Cancer Center, Houston, TX, United States; Department of Medicine, Memorial Sloan Kettering Cancer Center, New York, NY, United States; EMD Serono, Inc., Rockland, MA, United States, an affiliate of Merck KGaA; EMD Serono, Inc., Rockland, MA, United States, an affiliate of Merck KGaA; Princess Margaret Cancer Center, University of Toronto, Toronto, Ontario, Canada

**Keywords:** urinary bladder neoplasms, chemotherapy, immunotherapy, adverse effects, comorbidity

## Abstract

Platinum-based chemotherapy has been the standard first-line (1L) treatment for advanced urothelial carcinoma (UC) for decades, based on the proven efficacy and established safety profiles of cisplatin- and carboplatin-based regimens. With the emergence of novel regimens, it is important to reevaluate and contextualize the role of 1L platinum-based chemotherapy. Platinum-based chemotherapy followed by avelumab 1L maintenance in patients without disease progression following platinum-based chemotherapy was established as a standard 1L regimen based on the JAVELIN Bladder 100 phase III trial. More recently, the EV-302 phase III trial showed the superiority of 1L enfortumab vedotin (EV) + pembrolizumab versus platinum-based chemotherapy, and the Checkmate 901 phase III trial showed the superiority of 1L nivolumab + cisplatin/gemcitabine versus cisplatin/gemcitabine alone. These 2 regimens have now been included as standard 1L options in treatment guidelines for advanced UC. EV + pembrolizumab is now the preferred 1L treatment, and in locations where EV + pembrolizumab is not available or individual patients are not considered suitable, recommended options are platinum-based chemotherapy followed by avelumab maintenance or nivolumab + cisplatin-based chemotherapy. In this review, we discuss current treatment options for advanced UC recommended in guidelines, practical considerations with platinum-based chemotherapy, the role of avelumab 1L maintenance, recent phase III trials of EV + pembrolizumab and nivolumab + cisplatin/gemcitabine, safety profiles of recommended 1L treatments, and second-line treatment options.

## Teaser

This review reports currently recommended treatment options for advanced urothelial carcinoma, practical considerations with platinum-based chemotherapy, the role of avelumab first-line (1L) maintenance, recent phase III trials of enfortumab vedotin + pembrolizumab and nivolumab + cisplatin/gemcitabine, safety profiles of recommended 1L treatments, and 2L treatment options.

**Implications for Practice**

Enfortumab vedotin + pembrolizumab has superior efficacy versus platinum-based chemotherapy and is the preferred first-line (1L) treatment for advanced urothelial carcinoma. Understanding the distinct toxicity profile of enfortumab vedotin + pembrolizumab is an important consideration for daily practice. When enfortumab vedotin + pembrolizumab cannot be administered, cisplatin- and carboplatin-based regimens are alternative 1L options for cisplatin-eligible and cisplatin -ineligible patients. The efficacy of platinum-based chemotherapy is increased by administering immune checkpoint inhibitors in specific patient populations, including avelumab 1L maintenance treatment in patients who have stable disease or better with platinum-based chemotherapy, or nivolumab in combination with cisplatin and gemcitabine in cisplatin-eligible patients.

## Introduction

**Figure 1.**
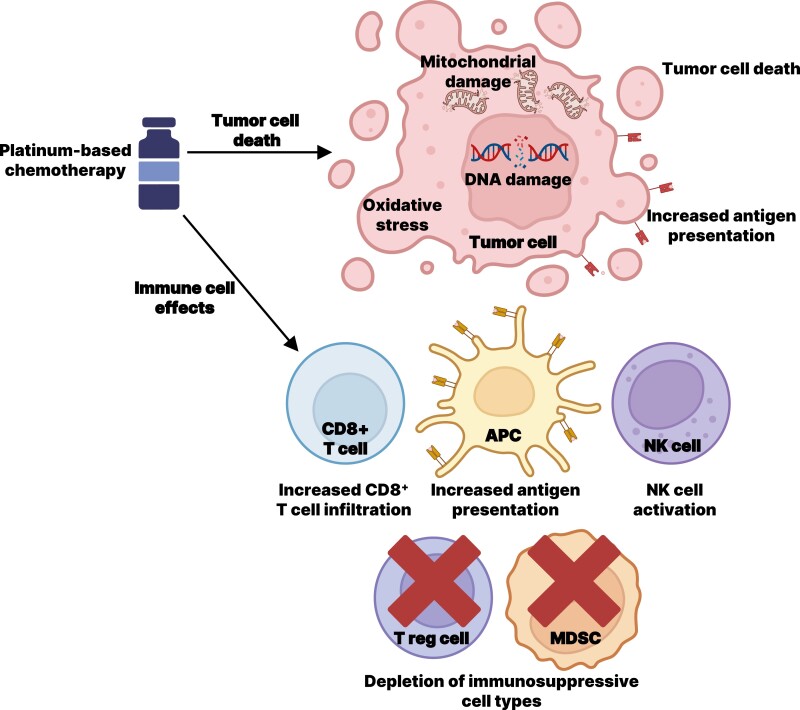
Antitumor mechanisms of platinum-based chemotherapy.^5-9^ Abbreviations: APC, antigen-presenting cell; MDSC, myeloid-derived suppressor cells; NK, natural killer.

Urothelial carcinoma (UC) is a chemotherapy-sensitive tumor, and platinum-based chemotherapy has been the cornerstone of first-line (1L) treatment for advanced UC for decades.^1-4^ The antitumor mechanisms of platinum-based chemotherapy include both direct cytotoxicity (DNA damage, mitochondrial damage, and oxidative stress) and potential immunogenic effects (Figure 1).^5-9^ Platinum agents have slightly different properties, with cisplatin having greater cytotoxicity and immunomodulatory activity than carboplatin.^8,10^ Mechanisms of resistance to platinum agents are complex and may include decreased uptake and apoptosis, and increased efflux, detoxification, and DNA repair.^11,12^

Avelumab, an immune checkpoint inhibitor (ICI), is the standard of care for 1L maintenance treatment in patients who receive 1L cisplatin- or carboplatin-based chemotherapy without disease progression (ie, stable disease [SD] or better).^1-4^ Approval was based on the JAVELIN Bladder 100 phase III trial, which showed that avelumab + best supportive care (BSC) significantly prolonged overall survival (OS) and progression-free survival (PFS) versus BSC alone in platinum-treated patients without disease progression.^13,14^

Three phase III trials of ICIs administered in combination with 1L platinum-based chemotherapy or as 1L monotherapy failed to show improved OS compared with platinum-based chemotherapy alone.^15-19^ However, in a sub-study of the CheckMate 901 phase III trial that enrolled cisplatin-eligible patients only, nivolumab in combination with 1L cisplatin/gemcitabine prolonged OS and PFS versus cisplatin/gemcitabine alone.^20^ This may reflect the increased activity of ICIs in combination with cisplatin versus carboplatin.^10,18^ In the EV-302 phase III trial, 1L enfortumab vedotin (EV) combined with pembrolizumab significantly improved OS and PFS versus 1L platinum-based chemotherapy.^21^ These results led to approvals of nivolumab + cisplatin/gemcitabine in cisplatin-eligible patients and enfortumab vedotin + pembrolizumab irrespective of cisplatin eligibility.^22-24^

With the evolution in treatments for advanced UC, it is important to reevaluate the role of 1L platinum-based chemotherapy as a treatment option. In this narrative review, we discuss current 1L treatment options according to international guidelines; practical considerations with platinum-based chemotherapy; phase III trials of avelumab 1L maintenance treatment, EV + pembrolizumab, nivolumab + cisplatin/gemcitabine, including safety profiles; and second-line (2L) treatment options.

## Recommended 1L treatment for patients with advanced UC

In updated international treatment guidelines, EV + pembrolizumab is the preferred 1L treatment for patients with advanced UC. Other 1L options are nivolumab + cisplatin/gemcitabine followed by nivolumab monotherapy or cisplatin-based chemotherapy followed by avelumab maintenance in patients without disease progression for cisplatin-eligible patients, or 1L gemcitabine + carboplatin followed by avelumab maintenance for cisplatin-ineligible patients.^1,2^ In patients unsuitable for any platinum-based chemotherapy, ICI monotherapy is a nonpreferred treatment option,^1-4^ with pembrolizumab approved in the United States.^25^ Previously, 1L ICI monotherapy with pembrolizumab or atezolizumab was approved for cisplatin-ineligible patients with programmed cell death 1 ligand 1 (PD-L1)+ tumors based on data from single-arm phase II trials^26,27^; however, following phase III trials showing no OS benefit versus platinum-based chemotherapy (IMvigor130 and KEYNOTE-361),^16-19^ US indications were withdrawn for atezolizumab and restricted to platinum-ineligible patients for pembrolizumab.^25,28^ In Europe and some countries outside of Europe, pembrolizumab and atezolizumab remain 1L options for cisplatin-ineligible patients with PD-L1+ tumors.^3,4^ In other countries, including Canada, ICIs are not an approved 1L option.^29^

## Eligibility for platinum-based chemotherapy

Clinical parameters guide whether patients are eligible for cisplatin-based chemotherapy, unsuitable for cisplatin but eligible for carboplatin-based chemotherapy, or unsuitable for any platinum-based chemotherapy.^1,3,4^ Ineligibility for cisplatin is generally based on consensus criteria reported by Galsky et al,^30,31^ alongside clinical judgment about the patient’s capacity to tolerate cisplatin treatment. The Galsky criteria include creatinine clearance (CrCl) of <60 mL/minute, Eastern Cooperative Oncology Group performance status (ECOG PS) of ≥2, hearing loss of grade ≥ 2, peripheral neuropathy of grade ≥ 2, and New York Heart Association heart failure of class III or IV. An estimated 30%-50% of patients with advanced UC are considered ineligible for cisplatin-based chemotherapy based on the Galsky criteria.^30^ However, to extend the use of cisplatin, a multidisciplinary approach has been suggested as an alternative to the absolute renal function threshold in patients with muscle-invasive UC. Consequently, in most academic centers, selected patients with borderline renal impairment (eg, CrCl 50-59 mL/minute) receive cisplatin-based chemotherapy with appropriate discussions and mitigation strategies.^32^ Additionally, to improve tolerability, cisplatin is frequently administered using a split-dose regimen (discussed later).

Criteria to identify patients ineligible for any platinum chemotherapy (cisplatin or carboplatin) have also been proposed. In a survey of 60 medical oncologists in the United States, the most commonly used criteria were ECOG PS of ≥3, CrCl of <30 mL/minute, peripheral neuropathy of grade ≥ 2, New York Heart Association heart failure of class ≥ III, or ECOG PS of 2 with CrCl of <30 mL/minute.^33^ Similarly, in the European Association of Urology guidelines, platinum ineligibility is defined as glomerular filtration rate of <30 mL/minute, ECOG PS of >2, ECOG PS of 2 with glomerular filtration rate of <60 mL/minute, or any comorbidities of grade > 2.^3^ Real-world studies of patients receiving 1L treatment for advanced UC before ICIs were available found that ≈60%-90% received platinum-based regimens.^34-36^ Similarly, in a study of medical records from 1868 European patients with metastatic UC receiving 1L treatment, 87% were considered eligible for platinum-based chemotherapy, including 55% eligible for cisplatin.^37^ In European surveys, renal function and ECOG PS were most commonly used to determine both platinum and cisplatin eligibility.^38^ Similarly, in a US survey, the most frequently considered factors when determining cisplatin ineligibility were renal dysfunction (78%) and poor PS (77%), followed by neuropathy (47%), solitary kidney (43%), hearing loss (43%), advanced age (43%), and cardiovascular dysfunction (41%).^39^ In recent clinical trials in cisplatin-ineligible populations, the most common reason for cisplatin ineligibility was renal impairment (49%-70% of patients), and other reasons included ECOG PS of 2 (10%-32%), concurrent renal impairment and ECOG PS of 2 (3%-9%), and hearing loss (≤15%).^26,27,40^

## Efficacy of platinum-based regimens in advanced UC

The efficacy of platinum-based chemotherapy and comparator regimens was established in several randomized trials (Table 1).^14-21,41-44^ An international phase III trial (*N* = 405) showed similar efficacy of gemcitabine + cisplatin versus standard-dose MVAC (methotrexate, vinblastine, doxorubicin, and cisplatin), including OS (median 14.0 vs 15.2 months; hazard ratio [HR], 1.09 [95% CI, 0.88-1.34]; *P* = .66) and PFS (median 7.7 vs 8.3 months; HR, 1.09 [95% CI, 0.89-1.34]; *P* = .63), whereas safety and tolerability were more favorable with gemcitabine + cisplatin.^41,45^ Subsequently, an international phase III trial (EORTC 30924; *N* = 263) found that OS and PFS were improved with dose-dense versus standard-dose MVAC (5-year OS rate, 21.8% vs 13.5%; HR, 0.76 [95% CI, 0.58-0.99]; *P* = .042; and median PFS, 9.5 versus 8.1 months; HR, 0.73 [95% CI, 0.56-0.95]; *P* = .017). The dose-dense regimen also resulted in a lower incidence of mucositis and leukopenia.^42^ In a randomized phase II trial of gemcitabine + cisplatin versus gemcitabine + carboplatin (*N* = 110), which was not powered to assess differences between arms, median time to progression was 8.3 versus 7.7 months, median OS was 12.8 versus 9.8 months, and objective response rate (ORR) was 66% versus 56%, respectively; no differences in toxicity rates were reported.^46^

**Table 1.** Median OS and PFS with 1L cisplatin- and carboplatin-based chemotherapy and comparator regimens in phase III trials. Abbreviations: 1L, first line; BSC, best supportive care; M-CAVI, methotrexate, carboplatin, and vinblastine; MVAC, methotrexate, vinblastine, doxorubicin, and cisplatin; OS, overall survival; PFS, progression-free survival.

Study	Regimen (number of patients)	OS, median, months	PFS, median, months
von der Maase^41^	Gemcitabine + cisplatin (*n* = 203)	14.0	7.7
	Non–dose-dense MVAC (*n* = 202)	15.2	8.3
EORTC 30924^42^	Dose-dense MVAC (*n* = 134)	15.1	9.5
	Non–dose-dense MVAC (*n* = 129)	14.9	8.1
EORTC 30986^43^	Gemcitabine + carboplatin (*n* = 110)	9.3	5.8
	M-CAVI (*n* = 108)	8.1	4.2
KEYNOTE-361^a^ (NCT02853305)^17^	Pembrolizumab + platinum-based chemotherapy (*n* = 351)	17.0	8.3
	Pembrolizumab monotherapy (*n* = 307)	15.6	Not reported
	Platinum-based chemotherapy (*n* = 352)	14.3	7.1
	Gemcitabine + cisplatin (*n* = 159)	Not reported	Not reported
	Gemcitabine + carboplatin (*n* = 196)	12.3	Not reported
IMvigor130^a^ (NCT02807636)^16,18,19^	Atezolizumab + platinum-based chemotherapy (*n* = 451)	16.0	8.2
	Atezolizumab monotherapy (*n* = 362)	15.2	Not reported
	Platinum-based chemotherapy (*n* = 400)^b^	13.3	6.3
	Gemcitabine + cisplatin (*n* = 136)^b^	13.4	6.4
	Gemcitabine + carboplatin (*n* = 264)^b^	13.4	6.3
DANUBE^a^ (NCT02516241)^15^	Durvalumab + tremelimumab (*n* = 342)	15.1	3.7
	Durvalumab monotherapy (*n* = 346)	13.2	2.3
	Platinum-based chemotherapy (*n* = 344)	12.1	6.7
CheckMate 901 (NCT03036098)^20^	Nivolumab + gemcitabine + cisplatin (*n* = 304)	21.7	7.9
	Gemcitabine + cisplatin (*n* = 304)	18.9	7.6
EV-302 (NCT04223856)^21^	Enfortumab vedotin + pembrolizumab (*n* = 442)	31.5	12.5
	Platinum-based chemotherapy (*n* = 444)	16.1	6.3
JAVELIN Bladder 100^‡^ (NCT02603432)^14,44^	Avelumab 1L maintenance (*n* = 350)^c^	23.8 (from start of maintenance)	5.5 (from start of maintenance)
	BSC (control arm; *n* = 350)^c^	15.0 (from start of maintenance)	2.1 (from start of maintenance)
	Platinum-based chemotherapy → avelumab 1L maintenance (*n* = 350)^c^	29.7	Not reported
	Gemcitabine + cisplatin → avelumab 1L maintenance (*n* = 183)^c^	31.0	Not reported
	Gemcitabine + carboplatin → avelumab 1L maintenance (*n* = 147)^c^	25.8	Not reported
	Platinum-based chemotherapy → BSC (*n* = 350)^c^	20.5	Not reported
	Gemcitabine + cisplatin → BSC (*n* = 206)^c^	23.0	Not reported
	Gemcitabine + carboplatin → BSC (*n* = 122)^c^	17.6	Not reported

^a^Trial did not meet its primary OS endpoint.

^b^Only the control arm population used for comparison with the atezolizumab + chemotherapy arm is reported.

^c^Trial population included only patients who did not have disease progression with 1L platinum-based chemotherapy.

Patients who are ineligible for cisplatin have been a focus of clinical trials based on a perception that carboplatin-based chemotherapy has inferior efficacy to cisplatin-based chemotherapy in advanced UC.^46-48^ However, patients considered unfit for cisplatin-based chemotherapy are generally older, have a worse PS, and are more likely to have comorbidities such as impaired renal function or heart failure compared with cisplatin-eligible patients, which may result in a worse prognosis irrespective of treatment.^30^ In general, 1L cisplatin-based chemotherapy is considered to have greater activity than carboplatin-based chemotherapy and is preferred versus carboplatin for eligible patients.^1,3,4^

## Safety considerations with platinum-based chemotherapy

Advanced UC occurs predominantly in older patients,^49^ who have an increased prevalence of renal dysfunction, reduced PS, and comorbidities, which are important factors when considering safety of treatment.^1,3,4,30^ Cisplatin- and carboplatin-based regimens have established safety profiles, and medical teams are familiar with managing associated adverse events (AEs). In patients with advanced UC, a maximum of 6 cycles of cisplatin- or carboplatin-based regimens is recommended, with fewer cycles acceptable in patients who have cumulative toxicity.^1,3,4^ In retrospective studies of patients with advanced UC who received platinum-based chemotherapy, 60%-67% of patients received ≥4 cycles (28%-46% receiving cisplatin-based chemotherapy and 48%-72% receiving carboplatin-based chemotherapy).^50-53^ Real-world studies suggest that OS in patients receiving 4 or ≥5 cycles of 1L platinum-based chemotherapy is similar.^54,55^

Common grade ≥ 3 AEs with platinum agents include myelosuppression/hematologic toxicity (grade ≥ 3 in 53%-72% of patients), gastrointestinal disorders (grade ≥ 3 in 3%-11%; eg, nausea, vomiting, constipation, and diarrhea), and fatigue/asthenia (grade ≥ 3 in 3%-11%).^17,20,56^ Myelosuppression may increase the risk of infection, fever, and bleeding complications.^57-60^ Standard hematologic tests should be performed before each cycle of cisplatin or carboplatin, and as clinically indicated. Patients should be closely monitored for the development of signs and symptoms of infection. In patients who develop severe myelosuppression, dose modifications and standard supportive care, such as transfusions and hematopoietic growth factors, should be considered. Cisplatin and carboplatin are highly and moderately emetogenic, respectively, and preventative antiemetic medication is often required.^57,58,60^

Cisplatin can cause renal toxicity, peripheral neuropathy, and ototoxicity, and it is generally avoided in patients with preexisting related comorbidities.^57-60^ To mitigate the potential for acute kidney injury, adequate hydration should be ensured before, during, and after cisplatin, and magnesium supplementation should be considered.^60^ During treatment, serum creatinine, blood urea nitrogen, creatinine clearance, and serum electrolytes should be monitored. Concomitant nephrotoxic medications, such as nonsteroidal anti-inflammatory drugs, diuretics, angiotensin-converting enzyme inhibitors, and angiotensin receptor blockers, should be minimized or discontinued before and during cisplatin treatment, if possible. To manage nephrotoxicity, neurotoxicity, and ototoxicity with cisplatin, patients may be switched to carboplatin or given a reduced dose/limited number of cycles.^32,57-60^ Gemcitabine + cisplatin is generally better tolerated than dose-dense MVAC; in the phase III trial of gemcitabine + cisplatin versus dose-dense MVAC as neoadjuvant treatment for muscle-invasive bladder cancer, gemcitabine + cisplatin resulted in significantly lower rates of grade ≥ 3 anemia (8% vs 22% of patients), nausea/vomiting (3% vs 10%), and asthenia (4% vs 14%).^56^

Additionally, split-dose cisplatin is a more tolerable alternative that results in reduced nephrotoxicity versus conventional single-day regimens, and is suitable for some patients considered cisplatin ineligible; however, efficacy data remain limited.^61-63^ A randomized phase II/III trial of split-dose cisplatin (35 mg/m^2^ on days 1 and 8 of 3-week cycles) + gemcitabine versus carboplatin + gemcitabine in patients with advanced UC and impaired renal function (CrCl 40-60 mL/minute) was stopped at interim analysis because of excess toxicity (predominantly renal toxicity) in the split-dose cisplatin arm,^64^ highlighting the importance of selecting suitable patients.^32^ More recently, an observational study in Germany showed comparable outcomes with split- versus standard-dose cisplatin administered in combination with gemcitabine.^65^ Larger prospective studies of split-dose cisplatin are needed.

## Avelumab 1L maintenance

Although 72%-79% of patients have an objective response or SD with 1L platinum-based chemotherapy alone, efficacy benefits are often not durable, with median duration of response of 6-8 months and median PFS of 6-7 months.^15-17^ In addition, across real-world studies in advanced UC, only 32%-52% of patients who received 1L platinum-based chemotherapy went on to receive 2L treatment, with low proportions receiving later lines.^50,66,67^ This high attrition rate highlights the aggressiveness of advanced UC and the rapid clinical deterioration that often accompanies disease progression. These observations provided the rationale for assessing avelumab 1L maintenance treatment, which builds on the clinical benefits of 1L platinum-based chemotherapy.

The JAVELIN Bladder 100 phase III trial enrolled patients with advanced UC who had completed 4-6 cycles of 1L gemcitabine + cisplatin or carboplatin without disease progression. Overall, 700 patients were randomized to receive avelumab 1L maintenance + BSC or BSC alone.^13,14^ After ≥ 2 years of follow-up in all patients, median OS (measured from randomization at the end of chemotherapy) was 23.8 versus 15.0 months, respectively (HR, 0.76 [95% CI, 0.63-0.91]; *P* = .0036). Median PFS was 5.5 versus 2.1 months, respectively (HR, 0.54 [95% CI, 0.46-0.64]; *P* < .0001).^14^ Median OS measured from start of 1L chemotherapy in this selected population was 29.7 months with avelumab + BSC versus 20.5 months with BSC alone.^44^ OS benefits with avelumab 1L maintenance were observed irrespective of 1L chemotherapy regimen (cisplatin- or carboplatin-based), best response to 1L chemotherapy (complete response, partial response, or SD), duration of 1L chemotherapy, receipt of 4 or 6 cycles of 1L chemotherapy, or PD-L1 status, suggesting that avelumab 1L maintenance is suitable for a broad range of patients and can be tailored according to practical considerations and patient needs.^13,14,44,68-70^

In safety analyses, 19.5% of patients had grade ≥ 3 treatment-related AEs (TRAEs); the most common TRAEs of any grade were pruritus (14.8% of patients; grade ≥ 3 in 0.3%), hypothyroidism (11.0%; grade ≥ 3 in 0.3%), fatigue (10.8%; grade ≥ 3 in 0.3%), and asthenia and diarrhea (10.5% each; grade ≥ 3 in 0%). In addition, immune-related AEs occurred in 32.3% of patients (grade ≥ 3 in 7.6%), and infusion-related reactions of any grade occurred in 21.8%. No new safety concerns were identified with long-term treatment.^14^ In US prescribing information, it is reported that 28% of patients treated with avelumab 1L maintenance had a serious adverse reaction, most commonly urinary tract infection (6.1%), pain (3.2%), and acute kidney injury (1.7%), and 0.3% had a fatal adverse reaction. Consistent with other ICIs, warnings and precautions are stated for immune-mediated adverse reactions (including pneumonitis, hepatitis, colitis, endocrinopathies, and nephritis/renal dysfunction), along with guidance for withholding or discontinuing treatment according to severity. Avelumab infusions should be interrupted or slowed in patients with mild/moderate infusion-related reactions or discontinued for severe infusion-related reactions.^71^

Overall, data from JAVELIN Bladder 100 support the recommendation of avelumab 1L maintenance in international treatment guidelines for both cisplatin-eligible and cisplatin-ineligible patients without disease progression after platinum-based chemotherapy.^1-4^

## First-line EV + pembrolizumab

EV + pembrolizumab is the preferred treatment for patients with advanced UC, irrespective of cisplatin eligibility.^1,2^ This regimen was initially evaluated in 2 populations of cisplatin-ineligible patients from the EV-103 phase Ib/II study: a single-arm population and a randomized population of patients treated with EV + pembrolizumab or EV alone.^40,72,73^ Subsequently, EV + pembrolizumab was compared with gemcitabine + cisplatin or carboplatin in 866 patients with advanced UC in the EV-302 phase III trial.^21^ In the EV + pembrolizumab arm, treatment was administered continuously until disease progression or unacceptable toxicity, with pembrolizumab administered for ≤35 cycles. In the chemotherapy arm, patients received a maximum of 6 cycles. After a median follow-up of 17.2 months, EV + pembrolizumab versus platinum-based chemotherapy resulted in significant improvements in OS (median 31.5 vs 16.1 months; HR, 0.47 [95% CI, 0.38-0.58]; *P* < .00001) and PFS (median 12.5 vs 6.3 months; HR, 0.45 [95% CI, 0.38-0.54]; *P* < .00001). The ORR in the EV + pembrolizumab arm versus the chemotherapy arm was 67.7% versus 44.4%, including complete response in 29.1% versus 12.5%, and the median duration of response was not reached versus 7.0 months, respectively. Subgroup analyses showed consistent efficacy benefits with EV + pembrolizumab versus chemotherapy across prespecified subgroups. In the chemotherapy arm, only 32.2% of patients received ICI maintenance (30.4% with avelumab), despite 78.2% achieving disease control with platinum-based chemotherapy. An additional 26.4% of patients in the chemotherapy arm received 2L ICI treatment after disease progression.^21^

In safety analyses, grade ≥ 3 TRAEs occurred in 55.9% of patients treated with EV + pembrolizumab versus 69.5% treated with chemotherapy, and serious TRAEs occurred in 27.7% versus 19.6%, respectively. Safety profiles were different between the treatment arms. In the EV + pembrolizumab arm, TRAEs of special interest included skin reactions (66.8% of patients; grade ≥ 3 in 15.5%), peripheral neuropathy (63.2%; grade ≥ 3 in 6.8%), and hyperglycemia (13.0%; grade ≥ 3 in 6.1%). In the chemotherapy arm, the most common TRAEs were anemia (56.6% of patients; grade ≥ 3 in 31.4%), neutropenia (41.6%; grade ≥ 3 in 30.0%), nausea (38.8%; grade ≥ 3 in 2.8%), fatigue (36.0%; grade ≥ 3 in 4.2%), and thrombocytopenia (34.2%; grade ≥ 3 in 19.4%).^21^ In US prescribing information, it is reported that 50% of patients treated with EV + pembrolizumab had a serious adverse reaction, most commonly rash (6%), acute kidney injury (5%), and pneumonitis/interstitial lung disease (4.5%), and 3.9% had a fatal adverse reaction.^22^ Prescribing information for EV includes warnings and precautions for hyperglycemia, pneumonitis/interstitial lung disease, peripheral neuropathy, ocular disorders, and infusion site extravasation, along with guidance for monitoring and adjusting or discontinuing treatment according to severity.^22^ Consistent with other ICIs, prescribing information for pembrolizumab includes warnings and precautions for immune-mediated adverse reactions (including pneumonitis, colitis, hepatitis, endocrinopathies, nephritis/renal dysfunction, and dermatologic adverse reactions) and infusion-related reactions.^25^ Prescribing information for EV includes a boxed warning about severe and fatal cutaneous adverse reactions, including Stevens-Johnson syndrome and toxic epidermal necrolysis, and also states that EV should be avoided in patients with moderate or severe hepatic impairment (Child-Pugh class B or C).^22^

Overall, results from the EV-302 trial demonstrated the efficacy and safety of 1L EV + pembrolizumab in patients with advanced UC, leading to its US approval in this population.^22^

## First-line nivolumab + cisplatin/gemcitabine

In a sub-study of the CheckMate 901 phase III trial, 608 cisplatin-eligible patients with advanced UC received nivolumab + cisplatin/gemcitabine followed by nivolumab monotherapy (maximum 24 months of nivolumab treatment) or cisplatin/gemcitabine only.^20^ After a median follow-up of 33.6 months, patients in the nivolumab + cisplatin/gemcitabine arm had significantly improved OS (median 21.7 vs 18.9 months; HR, 0.78 [95% CI, 0.63-0.96]; *P* = .02) and PFS (median 7.9 vs 7.6 months; HR, 0.72 [95% CI, 0.59-0.88]; *P* = .001) versus patients in the cisplatin/gemcitabine arm. ORRs in the nivolumab + cisplatin/gemcitabine and cisplatin/gemcitabine arms were 57.6% versus 43.1%, including complete response in 21.7% versus 11.8%, respectively. In patients who had an objective response, the median duration of response was 9.5 versus 7.3 months, and proportions with an ongoing response at 2 years (maximum duration of study treatment) were 35.0% versus 12.6%, respectively.^20^ Median duration of complete response was 37.1 versus 13.2 months.^20,74^ In the chemotherapy alone arm, only 20% of patients received ICI maintenance therapy, despite 71.4% achieving disease control.

In safety analyses, grade ≥ 3 TRAEs occurred in 61.8% of patients treated with nivolumab + cisplatin/gemcitabine versus 51.7% treated with cisplatin/gemcitabine. The most common TRAEs with nivolumab + cisplatin/gemcitabine versus cisplatin/gemcitabine were anemia (57.2% vs 47.6% of patients; grade ≥ 3 in 22.0% vs 17.7%), nausea (46.7% vs 47.9%; grade ≥ 3 in 0.3% vs 1.0%), neutropenia (30.6% vs 29.9%; grade ≥ 3 in 18.8% vs 15.3%), and decreased neutrophil count (24.7% vs 20.8%; grade ≥ 3 in 14.5% vs 11.1%), respectively.^20^ Consistent with other ICIs, US prescribing information for nivolumab includes warnings and precautions for immune-mediated adverse reactions, including pneumonitis, colitis, hepatitis, endocrinopathies, dermatologic adverse reactions, and nephritis/renal dysfunction, in addition to infusion-related reactions.^23^

Overall, CheckMate 901 was the first phase III trial to show an OS benefit with an ICI administered concurrently with chemotherapy (specifically cisplatin-based chemotherapy) in its primary analysis, and its findings supported the US and European approval of nivolumab + gemcitabine/cisplatin for cisplatin-eligible patients with advanced UC.^23,24^

## Second-line treatment

Several treatment options are available for 2L or later treatment of advanced UC, although options vary based on the 1L treatment received.^1-3^

In patients who have received 1L platinum-based chemotherapy followed by avelumab 1L maintenance or 1L nivolumab + cisplatin/gemcitabine, subsequent treatment options include EV (based on the EV-301 phase III trial^75^), erdafitinib (in patients with *FGFR3* alterations, based on the THOR phase III trial^76^), sacituzumab govitecan (ADC targeted to trophoblast cell-surface antigen 2, based on the TROPHY-U-01 phase II study^77^), as well as platinum rechallenge and nonplatinum chemotherapy (eg, taxanes).^1-4^ It has recently been announced that the TROPiCS-04 phase III trial, which compared sacituzumab govitecan versus physician’s choice of single-agent chemotherapy in patients with prior platinum-based chemotherapy and PD-(L)1 inhibitor treatment, did not meet its primary OS endpoint; it is currently unclear how this will impact its usage.^78^ In the JAVELIN Bladder 100 trial, a high proportion of patients received 2L treatment after avelumab (60% of patients who discontinued for any reason and 76% who discontinued due to disease progression). Most patients received chemotherapy, and only 2.9% of those who discontinued avelumab received 2L EV, reflecting available options when the study was conducted.^14,79^ However, in a real-world study of patients with advanced UC who received avelumab 1L maintenance in the United States, 70% of those who received 2L treatment received EV.^80,81^ Real-world studies have demonstrated that OS durations with 2L EV in patients who have had disease progression following 1L platinum-based chemotherapy and avelumab 1L maintenance are consistent with observations in the EV-301 trial.^81,82^ Furthermore, in a real-world study performed in France, the median OS from the start of avelumab 1L maintenance treatment was 31.3 months in 52 patients who subsequently received 2L ADC treatment (EV in 46 patients) compared with 14.0 months in 238 patients who received 2L chemotherapy.^81^

For patients who have disease progression during 1L platinum-based chemotherapy, preferred 2L therapy is pembrolizumab (based on phase III data)^83^; alternatives (in the United States) include other ICIs (nivolumab or avelumab; based on single-arm data), in addition to EV, erdafitinib, and nonplatinum chemotherapy, as noted above.^1-4^

For patients who have received 1L EV + pembrolizumab, subsequent treatment is an important area that requires further study.^84^ Potential options suggested in recently updated treatment guidelines are platinum-based chemotherapy (without maintenance) or erdafitinib (patients with *FGFR3* alterations only).^1,2^ In the EV-302 trial, among patients who received subsequent treatment after EV + pembrolizumab (29% of the treatment arm), most (25%) received platinum-based chemotherapy^21^; however, data to demonstrate efficacy and safety are needed. Regarding the use of erdafitinib or sacituzumab govitecan as later-line treatment, clinical studies enrolled only patients who had received prior platinum-based chemotherapy, and data in patients who received prior EV + pembrolizumab treatment are currently lacking. Data showing long-term outcomes with different treatment sequences are needed.

## Discussion

The treatment landscape for advanced UC has evolved rapidly. Following the results of the EV-302 phase III trial, EV + pembrolizumab has become the preferred 1L treatment option, irrespective of cisplatin eligibility.^1,2^ EV + pembrolizumab was approved in the United States in December 2023. Although cross-trial comparisons are challenging, EV + pembrolizumab appears to provide the greatest overall efficacy benefit versus standard platinum-based chemotherapy reported to date. Education of clinical teams and patients will be needed to help manage the toxicities specifically associated with EV + pembrolizumab, including neuropathy, skin reactions, and hyperglycemia. Early toxicity recognition and intervention are needed, and subspecialty care may be necessary for co-management of toxicities.^85,86^ Patients with uncontrolled diabetes or baseline neuropathy may not be candidates for EV + pembrolizumab based on clinical judgment.

When EV + pembrolizumab is not available, or where toxicity risks associated with EV + pembrolizumab are felt to outweigh potential efficacy benefits in individual patients, other recommended 1L options are nivolumab plus cisplatin/gemcitabine (in cisplatin-eligible patients only; based on the CheckMate 901 trial) or platinum-based chemotherapy followed by avelumab 1L maintenance (based on the JAVELIN Bladder 100 trial).^1,2^ Cisplatin-based chemotherapy is preferred over carboplatin-based chemotherapy, and split-dose cisplatin is a potential option with improved tolerability compared with conventional cisplatin dosing in borderline cisplatin-eligible patients. Opinions about the risk:benefit ratio with EV + pembrolizumab versus other options are likely to vary and may depend on fitness and comorbidities in individual patients.^87-89^ Additionally, recent data suggest that patients with a low tumor burden (eg, nonvisceral or lymph node-only disease) have a good overall prognosis and have favorable outcomes with all recommended 1L options.^90-92^

Several outstanding questions remain, particularly optimal treatment sequences with available options after 1L treatment. It is also unclear whether prior exposure to neoadjuvant or adjuvant treatment, which may include cisplatin-based chemotherapy or ICI treatment, affects choice and outcomes with current 1L options. In recent trials of current 1L options, eligibility criteria specified that patients could not have received neoadjuvant or adjuvant treatment within the prior 12 months.^13,20,21^ Novel biomarkers to help guide treatment selection with different 1L options are urgently needed.^93^

## Conclusion

Overall, it is an exciting time in the UC field with many recent developments, especially in the 1L setting. Although EV + pembrolizumab has become a preferred option in the US, platinum-based chemotherapy remains an important treatment for many patients with advanced UC globally. A key issue is to ensure access to new treatment options, both upfront and sequentially, to maximize overall patient outcomes.

## Data Availability

No new data were generated or analyzed during the preparation of this manuscript.
